# A prospective, non-randomized evaluation of a novel low energy radiofrequency treatment for nasal obstruction and snoring

**DOI:** 10.1007/s00405-018-05270-y

**Published:** 2019-01-03

**Authors:** Detlef Brehmer, Robert Bodlaj, Friedemann Gerhards

**Affiliations:** 1 Faculty of Medicine, University Witten / Herdecke, Alfred-Herrhausen-Straße 50, 58455 Witten, Germany; 2 Department of Electrical Engineering and Applied Natural Sciences, Westphalian University of Applied Sciences, Neidenburger Str. 43, 45897 Gelsenkirchen, Germany; 3 Department of Otorhinolaryngology, Private ENT Practice, Friedrichstr. 3/4, 37073 Goettingen, Germany; 4 ENT Practice Lichtenfels, Bamberger Straße 7, 96215 Lichtenfels, Germany; 5 Center for Psychobiological and Psychosomatic Research, Trier University, Johanniterufer 15, 54290 Trier, Germany

**Keywords:** Nasal valve, Radiofrequency therapy, Snoring, Nose score

## Abstract

**Background:**

Weak or inward-bent cartilage of the nasal sidewall at the level of the internal nasal valve (INV) can produce narrowness or collapse of the nasal valve. This is a common cause of impaired nasal breathing during daily activities and there is also an established connection between nasal obstruction and snoring. The condition is often difficult to treat, although even a small enlargement of the lumen at the nasal valve can lead to a significant improvement in the ease of nasal breathing.

**Methods:**

The primary objective of this prospective study was to evaluate the safety and efficacy of the Vivaer system for the treatment of narrowed nasal valves and to measure changes in the symptoms of nasal obstruction and snoring. The Vivaer system uses low energy radiofrequency to remodel the nasal sidewall in order to improve airflow.

**Results:**

The study involved 31 patients presenting from 1st September 2017 to 1st May 2018 with symptoms of nasal obstruction and snoring. In all patients, an improvement was observed in nasal breathing measured by NOSE score, sleep quality by SOS questionnaire and quality of life as measured by EQ-5D and SNOT-22.

**Conclusion:**

Vivaer intranasal remodeling can provide a durable and well-tolerated non-invasive treatment for those patients who are suffering congestion due to narrowness or collapse of the INV.

## Introduction

Impaired nasal breathing is a common reason for patients’ consultations to the ENT physician and is considered by those affected to cause significant reduction in their quality of life [1]. Habitual snoring can have an incidence of up to 50% and can be a serious social problem for the patient and the bed partner [2]. Amongst anatomical causes, septal deviation plays an important role in many patients. Even so, a combined septoplasty with turbinate reduction does not always provide a complete solution to the problem. The most frequent cause of septoplasty failure is closely related to the nasal valve [3].

The Vivaer intranasal remodeling treatment is a minimally invasive procedure and uses a stylus to deliver controlled and targeted low energy radiofrequency heating to the nasal sidewall to gently reshape the tissues. Unlike most established treatments, it is an outpatient intervention administered under local anesthesia.

In this study, we aim to investigate the effect of using isolated intranasal remodeling of the INV on measures of nasal breathing and snoring.

## Materials and methods

### Participants and setting

**Fig. 1:**
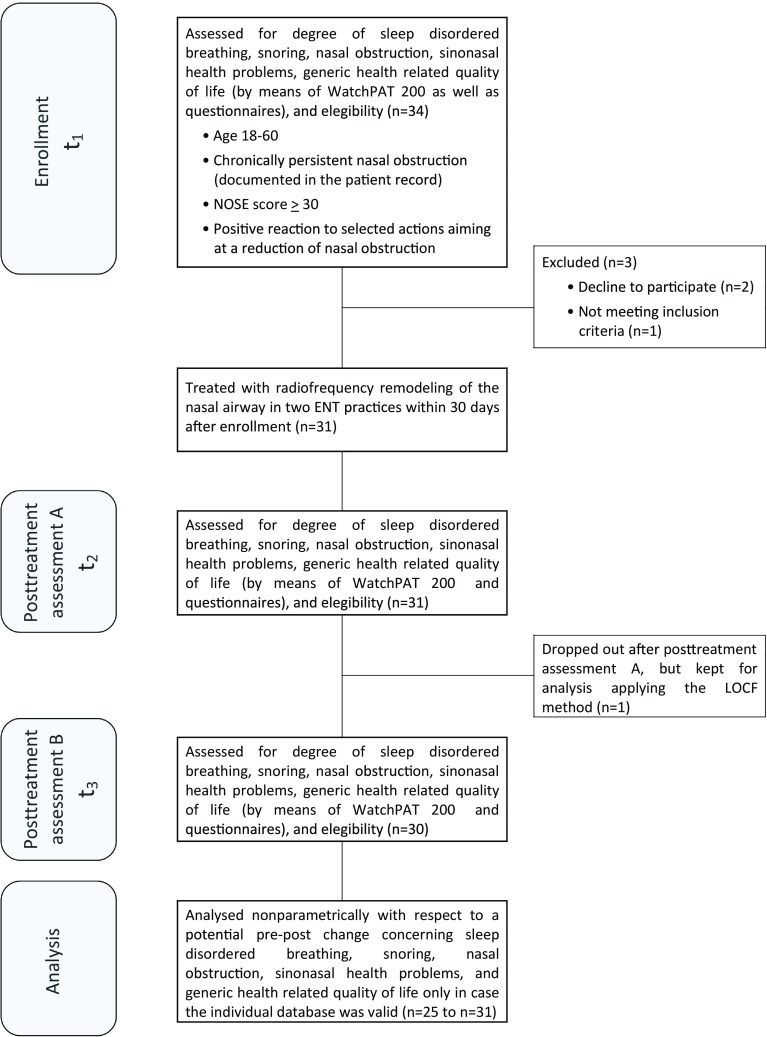
Schematic diagram summarizing the study design. WatchPAT200: wrist-worn device measuring peripheral arterial tone. *NOSE* nasal obstruction symptom evaluation scale, *LOCF* last observation carried forward

This study was a prospective, uncontrolled open-label bicenter trial which was conducted from September, 2017 to June, 2018 in ENT practice Göttingen and ENT practice Lichtenfels. The flowchart diagram demonstrates the study design (Fig. 1).

Thirty-four patients reporting snoring during sleep due to nasal obstruction or problems breathing through the nose were prospectively recruited for low energy radiofrequency remodeling treatment of the nasal valve.

The assessments by means of the questionnaires listed below and the WatchPAT200 device were conducted at study enrollment (measurement time point *t*_1_) as well as 90 days after the radiofrequency treatment (measurement time point *t*_3_). Thirty days after the treatment (measurement time point *t*_2_) patients filled in the two questionnaires measuring nasal obstruction and snoring (NOSE, SOS). The treatment was conducted within 30 days after the study enrollment. The patients´ satisfaction with the treatment was assessed 90 days after the intervention by means of a 10-point Likert scale (1 = completely dissatisfied; 10 = very satisfied).

At study enrollment, several inclusion and exclusion criteria were used. The inclusion criteria for a patient to participate were: (a) age, 18–60 years; (b) a chronically persistent nasal obstruction documented in the patient record; (c) a positive reaction to a minimum of two of four diagnostic tests for nasal obstruction at the INV (1. application of dilatative nasal strips like breathe right; 2. modified Cottle test using a Q-tip; 3. use of nasal stents; 4. Cottle test); (d) a minimum score of 30 in the Nasal Obstruction Symptom Evaluation (NOSE) Scale. The NOSE survey consists of five items assessing problems associated with or resulting from nasal obstruction [4]. A NOSE score of 30 is most appropriate to differentiate patients with and without nasal obstruction and is indicative of at least a moderate (instead of only mild) severity of nasal obstruction according to findings of Lipan and Most [5].

Patients were excluded if they (a) had a prior surgery of the nasal valve; (b) snored due to an abnormal anatomy of the palate, uvula or tonsil function; (c) had a body mass index, ≥ 40 kg/m^2^.

*N* = 3 patients were excluded due to ‘Not meeting inclusion criteria’ or ‘Declined to participate’. Therefore, 31 participants (17 females, 14 males) were included in the study. Their mean age was 42.84 years (SD = 11.27; range 24–64) and their mean BMI was 26.18 (SD = 4.30; range 20–38).

The study was carried out according to the principles stated in the 1964 Declaration of Helsinki and its later amendments. The study was approved by the Ethics Commission at the University of Witten/Herdecke, Germany. Once written patient consent was obtained patients received a standardized nasal examination (see above) and endoscopy and paper preoperative questionnaires. In each study center, a study nurse was in charge of the data collection.

### Evaluation of treatment success by means of data from questionnaires and sleep studies

For an evaluation of treatment success, we used questionnaires as well as data from sleep studies (see below). Assessments for the evaluation were conducted at study enrollment (measurement time point *t*_1_) as well as 30 and 90 days after the radiofrequency treatment (post-treatment time points *t*_2_ and *t*_3_). Patients were discharged from the study after they had participated in the last posttreatment assessments (*t*_3_).

The questionnaires used assess problems associated with or resulting from nasal obstruction and snoring, sinonasal health problems, and generic health-related quality of life. To incorporate only valid information, a sum or mean questionnaire score was calculated only if at least 75% of the items of a questionnaire had been answered.

For the assessment of subjective nasal airway obstruction, we used the NOSE which has been shown to be a valid and reliable instrument [4]. The NOSE survey has been constructed especially for the use in outcome studies in adults with nasal obstruction [4]. Each of the NOSE items is scored using a 5-point Likert scale to make a total score range of 0 through 100. Higher scores indicate worse obstruction. Studies reporting outcomes using the NOSE scale often demonstrate improvement after surgical treatment [6].

To assess subjective snoring severity, we selected four items (#1, #3, #7, #8) from the Snore Outcomes Survey (SOS) developed by Gliklich and Wang [7] with the intention to measure snoring and sleep-disordered breathing. The selected items focus on (a) the amount of sleeping time spent snoring; (b) the extent of awakening due to snoring and/or sleepiness due to poor sleep; and (c) the sound intensity of snoring. In case a patient answered a question with “I don’t know” the answer was rated as “missing”. Answers were coded from 0 (lowest degree of severity) to 3 or 4 (highest degree of severity) depending on the number of response levels per item. By dividing the achieved severity score by the maximal score and by multiplying this division by 100, all answers were transformed into a uniform response format with scores ranging from 0 to 100. We checked the reliability of our instrument on the basis of the data from this study (see Data Analyses and Results).

As a measure of health-related quality of life that was constructed specifically for patients with sinonasal health problems, we used the SNOT-20-GAV [8–10]. This instrument is a further German development (GAV = German Adapted Version) of the Sino-Nasal Outcome Test 20 constructed by Piccirilo et al. [11]. The SNOT-20-GAV proved to be valid and reliable, it allows for the calculation of a total score (TS) as well as of subscale scores representing primary nasal symptoms (PNS), secondary rhinogenic symptoms (SRS) and quality of life in general (QLG). Each score ranges from 0 to 100, higher scores indicate worse health or quality of health.

Generic quality of life was furthermore assessed by means of the EQ-5D. This instrument was designed by the EuroQoL group [12], it consists of a visual analog scale and five items (concerning mobility, self-care, usual activities, pain/discomfort and anxiety/depression) with five response levels each. With regard to the evaluation of the five items, we applied a method presented by Hinz et al. who calculated an individual crude sum score that was transformed into a score ranging from 0 to 100 indicating low-to-high quality of life [12]. Hinz et al. showed that this simple form of evaluation yielded values that were highly correlated (*r* = 0.93) with those of the far more complex conventional measurement [13].

The NOSE and the selected SOS items were administered at all three assessment time points, the remaining questionnaires were filled in by the patients at time points *t*_1_and *t*_3_ (see Fig. 1).

At study enrollment (time point *t*_1_) as well as 90 days after the treatment (time point *t*_3_), every patient participated in a home sleep study making use of the portable wrist-worn diagnostic device WatchPat200 (Itamar Medical Ltd, 3579 Caesarea, Israel). This device measures pulse oximetry and peripheral arterial tone (PAT) and allows for a reliable assessment of relevant indicators of sleep-disordered breathing. Different studies show a high correlation of results from simultaneous recordings of WatchPAT200 and polysomnography [14, 15]. The WatchPat 200 is FDA-approved and has a Conformitè Europeene (CE) mark. As a central parameter of sleep-disordered breathing, we determined the Apnea–Hypopnoea Index (AHI). We furthermore used the WatchPAT to assess the snoring volume by means of its integrated acoustic decibel detector and determined the mean snoring volume during sleep as well as the percentage of sleeping time spent snoring with an intensity above 45 dB.

One patient dropped out after the assessment at time point *t*_2_. For the purpose of our data analyses, the missing data of this patient concerning the measurement 90 days after treatment (*t*_3_) were filled in according to the LOCF-method (LOCF: last observation carried forward).

### Procedural technique

The treatment was conducted within 30 days after the study enrollment. All procedures were performed by two of the three authors (DB and RB), one surgeon per patient was involved. And neither of them participated in the collection of the questionnaires.

**Fig. 2:**
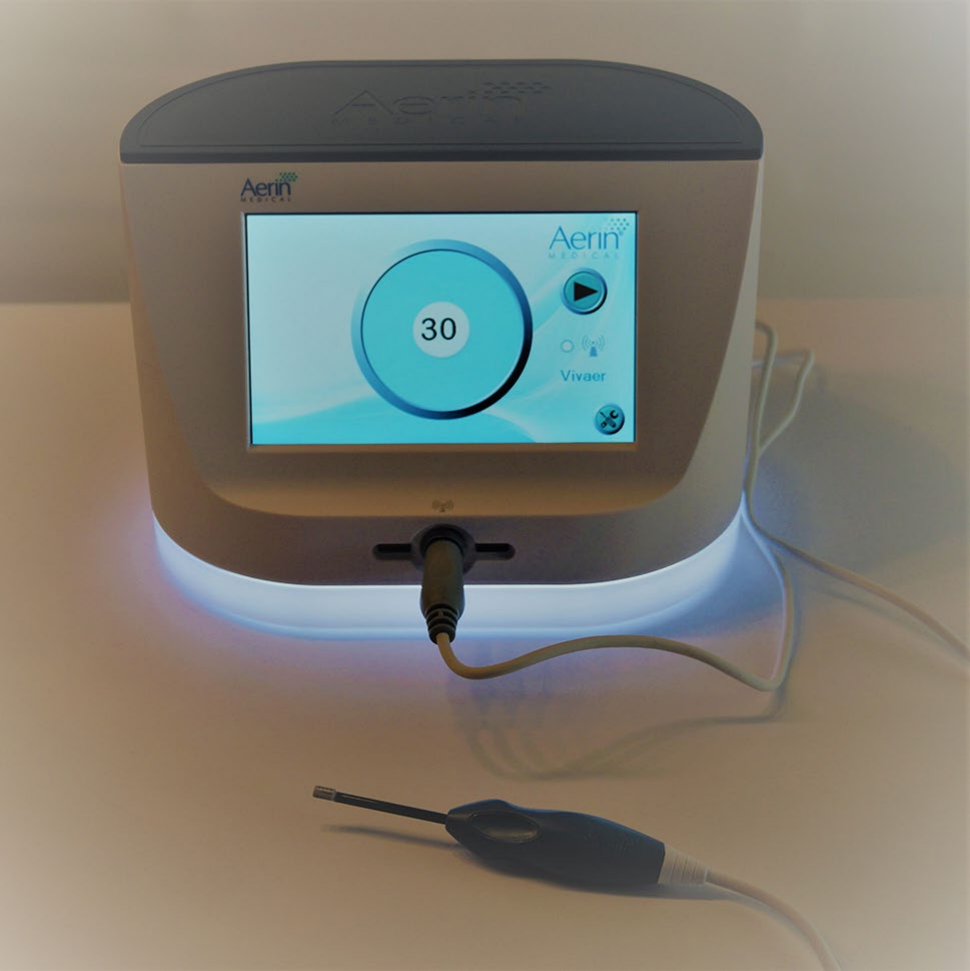
The RF remodeling platform

**Fig. 3:**
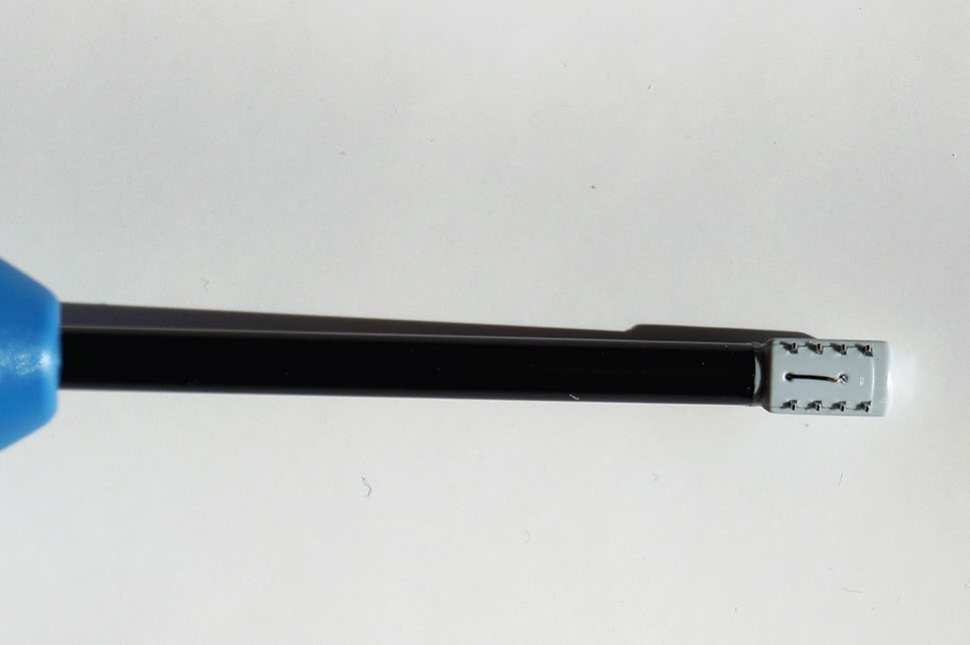
Handpiece treatment device

The nasal valve regions of all patients were treated bilaterally on an outpatient basis under local anesthesia using a handheld single-use device, the Vivaer™ stylus (Aerin Medical Inc. Sunnyvale, CA 94089 USA). The device consists of a handle, shaft and treatment tip and is designed to be used with the Aerin console to deliver temperature controlled, low energy radiofrequency to the target tissue (Fig. 2). An array of bipolar electrodes at the treatment tip is designed to deform the nasal valve into a curve whilst delivering bipolar energy into the tissue (Fig. 3). A temperature sensor at the tip ensures a constant, low temperature is maintained through the treatment. The device has been awarded a Conformité Européene (CE) mark and thus meets the requirements of the EU guidelines on health and safety.

**Fig. 4:**
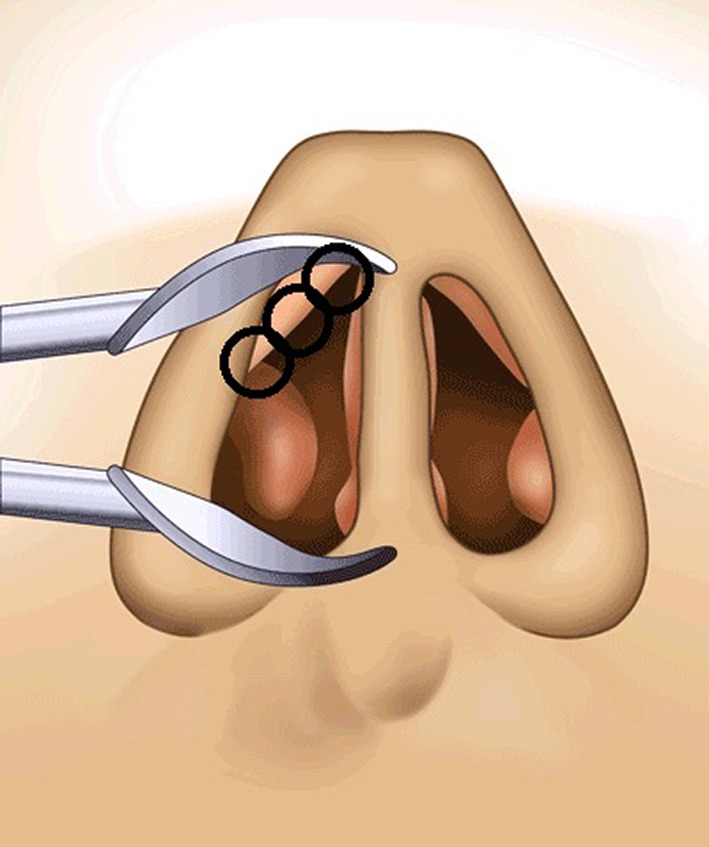
Treatment points

The treatment criteria used were: temperature 60 °C, maximum power 4 W, treatment duration 18 s, and cooling time 12 s. After the application of a submucosal local anesthetic along the scrolling edge of the upper lateral cartilage of both nasal valve regions, the treatment tip of the stylus was inserted into the nostril and pressed against the mucosa of the caudal region of the ULC. The surgeon administered the 30 s treatment, whilst deflecting the nasal wall laterally. This procedure was carried out 3 (maximum 4) times per nostril on adjacent areas of tissue at the ULC (Fig. 4). No special post-treatment actions were performed although patients were instructed to refrain from any manipulation of the treatment area.

The follow-up phase began after the procedure. Patients underwent post treatment visits with completion of the questionnaires after 30 and 90 days (as usual for this procedure). Requirements for each follow-up visit are listed in Table 1. Patients were discharged from the study on the 90 day follow-up visit.

**Table 1:** Medium score (and interquartile range) for the Nasal Obstruction Symptom Evaluation Scale (NOSE) and the Snore Outcomes Survey (SOS; selected items) at study enrollment (*t*_1_) and 30 days after radiofrequency treatment (*t*_2_); sample size and significance level of the Wilcoxon signed-rank test

	*t*_1_	*t*_2_	*N*	*p*_(one-tailed)_
	Mdn (IQR)	Mdn (IQR)		
Nasal Obstruction Symtom Evaluation Scale (NOSE)	65.00 (21.25)	30.00 (35.00)	30	**0.000**
Snore Outcomes Survey (SOS; selected items)	66.67 (32.29)	47.92 (40.63)	25	**0.000**

Bold—Significance level *p*: probability that the difference between the data collected at the measurement time points mentioned occured randomly or by chance, but is instead attributed to the low energy radiofrequency remodeling treatment (probabilty of error α)

Statistical comparisons by Wilcoxon signed-rank test; *p* significance level (one-tailed) concerning the difference of scores at time points *t*_1_ and *t*_2_

*Mdn* median, *IQR* interquartile range, *N* number of participants

Each patient´s satisfaction with the treatment was assessed 90 days after the radiofrequency treatment by means of a 10-point Likert scale (1 = completely dissatisfied; 10 = very satisfied).

## Data analyses and results

Data were analyzed using IBM SPSS Statistics, Version 23.0 (IBM SPSS Inc., Chicago, USA). The analysis of patients’ ratings of satisfaction with the treatment showed that nearly 76% of the sample submitted satisfaction ratings located in the upper half of the Likert scale (range 6–10); the modal value was 8 (median = 7). These results indicate a high level of contentment with the radiofrequency treatment in ¾ of the patients.

Because Shapiro–Wilk tests of normality showed that not all dependent variables regarded as indicators of treatment success were normally distributed at each measurement time point we decided to analyze all data uniformly by Wilcoxon signed-rank tests. This non-parametric way of data analysis is recommended as the better choice in non-normal situations compared to Student’s *t* test, the power advantages of which are small even under normal theory [16, 17].

First of all, we checked the reliability of our questionnaire assessing snoring severity (selected SOS items) by the calculation of Cronbach’s alpha for each of the three measurement time points. The α coefficients ranged from 0.81 to 0.90, indicating a good internal consistency of our instrument.

We secondly analyzed whether patients’ answers to the NOSE items and the selected SOS items at study enrollment (*t*_1_) were different from their answers 30 days after radiofrequency treatment (*t*_2_). Our analyses revealed (see Table 1) that 1 month after treatment there was a highly significant (*p* < 0.000) reduction of problems associated with or resulting from nasal obstruction (NOSE) as well as of snoring and problems due to snoring/poor sleep (SOS).

**Fig. 5:**
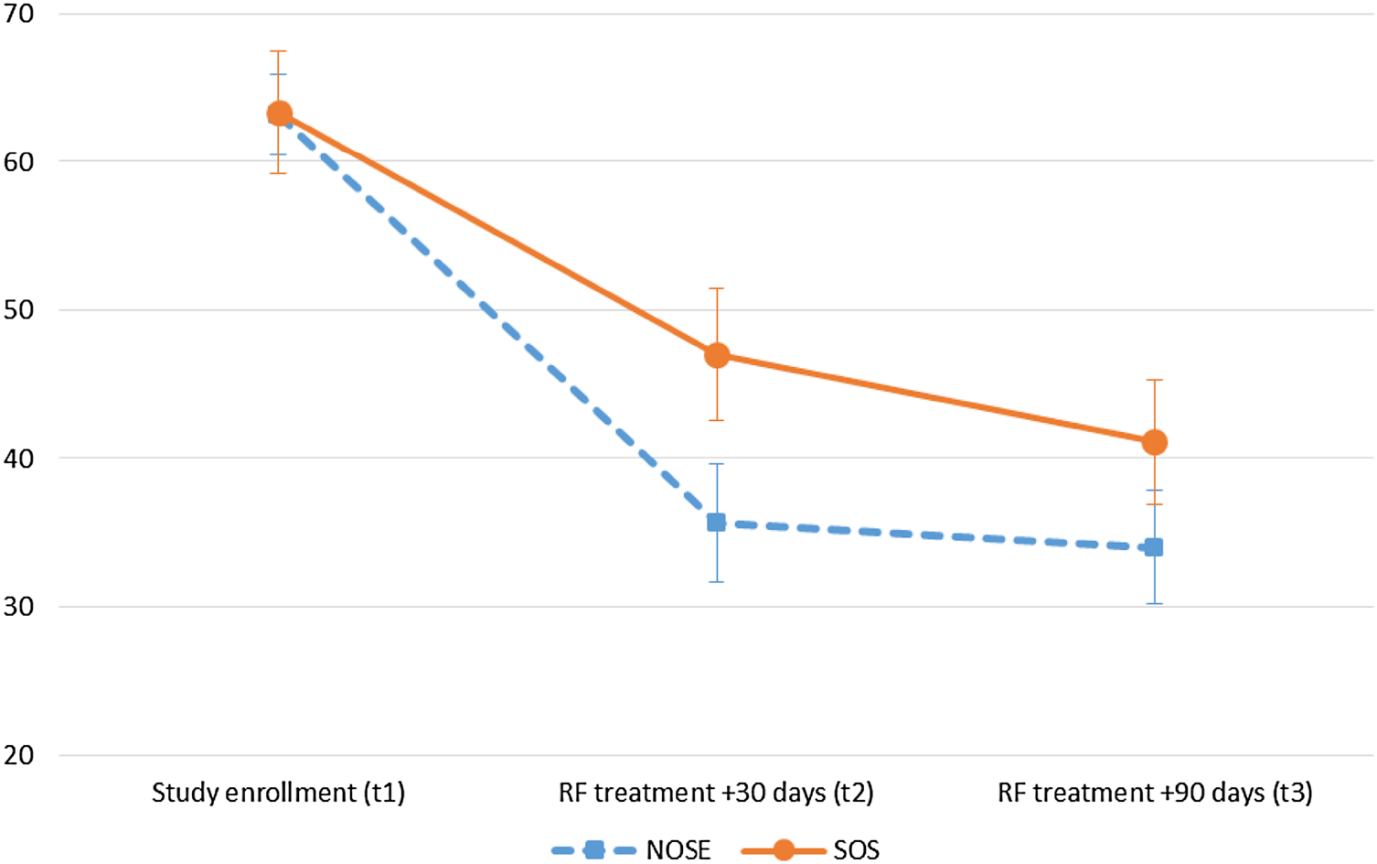
Development of nasal obstruction and snoring: mean (± SEM) of the NOSE and SOS score at pre- and post-treatment assessment time points. *NOSE* Nasal Obstruction Symptom Evaluation Scale, *SOS* Snore Outcomes Survey (selected items), *RF* radiofrequency

The comparison of the NOSE and SOS data collected at study enrollment (*t*_1_) and 90 days after treatment (*t*_3_) showed that 3 months after surgery there was still a highly significant reduction (p < 0.000; see Table 2) of problems concerning nasal obstruction and snoring. Figure 5 shows the change over time for both variables.

**Table 2:** Medium score (and interquartile range) for the Nasal Obstruction Symptom Evaluation Scale (NOSE), the Snore Outcomes Survey (SOS; selected items), the 20-Item Sino-Nasal Outcome Test, German Adapted Version (SNOT-20 GAV), the EQ-5D-5L questionnaire and parameters of disordered breathing and snoring during sleep assessed by the WatchPAT200 at study enrollment (*t*_1_) and 90 days after radiofrequency treatment (*t*_3_); sample size and significance level of the Wilcoxon signed-rank test

	*t*_1_	*t*_3_	*N*	*p*_(one-tailed)_
	Mdn (IQR)	Mdn (IQR)		
Nasal Obstruction Symtom Evaluation Scale (NOSE)	65.00 (20.00)	30.00 (25.00)	31	**0.000**
Snore Outcomes Survey (SOS; selected items)	66.67 (31.25)	41.67 (29.17)	27	**0.000**
20-Item Sino-Nasal Outcome Test, German Adapted Version (SNOT-20 GAV)				
Total Score (TS)	30.00 (26.00)	19.00 (16.00)	31	**0.000**
Primary Nasal Symptoms (PNS)	32.00 (28.00)	20.00 (16.00)	31	**0.000**
Secondary Rhinogenic Symptoms (SRS)	20.00 (20.00)	10.00 (10.00)	31	**0.000**
Generic Quality of Life (GQL)	28.89 (28.89)	20.00 (24.44)	31	**0.003**
EuroQoL group questionnaire for generic quality of life (EQ-5D-5L)				
Visual Analog Scale (VAS)	80.00 (22.50)	80.00 (21.00)	29	**0.030**
Sum Score (SS)	95.00 (20.00)	95.00 (10.00)	31	**0.033**
WatchPAT200				
Apnea–Hypopnea Index (AHI)	8.21 (10.39)	8.15 (16.96)	31	0.197
Mean snoring intensity (dB)	41.00 (1.00)	41.00 (1.00)	30	0.497
Percent of sleeping time spent with a snoring intensity > 45 dB	2.25 (5.18)	2.45 (5.13)	30	0.237

Bold—Significance level *p*: probability that the difference between the data collected at the measurement time points mentioned occured randomly or by chance, but is instead attributed to the low energy radiofrequency remodeling treatment (probabilty of error α)

Statistical comparisons by Wilcoxon signed-rank test; *p* significance level (one-tailed) concerning the difference of scores at time points *t*_1_ and *t*_3_

*Mdn* median, *IQR* interquartile range, *N* number of participants

In addition to analyzing treatment success of the total patient group, we determined the magnitude of long-term nasal obstruction change in terms of percentage improvement for patient subgroups with distinct pre-treatment nasal obstruction severity levels. Lipan and Most [4] proposed a severity classification system for nasal obstruction that comprises four classes of severity: the obstruction severity in patients who achieve a NOSE score of 5–25 is graded “mild”, the grades above are labeled “moderate” (30–50), “Severe” (55–75), and “extreme” (80–100). In our study, the patients with an initial “moderate” nasal obstruction (*n* = 7) showed a medium percentage of the NOSE score reduction of 37.50% 3 months after treatment (*t*_3_), those with an initial “severe” obstruction (*n* = 18) had a reduction of 37.86%. Patients whose initial obstruction was graded “extreme” (*n* = 6) presented with an improvement of 69.30%. We also looked at improvement in terms of a patient´s potential down-stepping to a lower nasal obstruction severity level. Our cross-tabulation of severity classes at study enrollment (*t*_1_) and 90 days after treatment (*t*_3_) did not show any class-change over time in the negative direction. A continuance in the same severity class was observed in *n* = 6 cases, whereas a positive change of one grade was seen in *n* = 15 cases. The remaining cases presented with a positive change of two (*n* = 7) or even three (*n* = 3) grades. In 25 of 31 patients, i.e., in > 80%, there was an improvement.

Further Wilcoxon signed-rank tests of the patients’ answers at study enrollment (*t*_1_) and 90 days after treatment (*t*_3_) concerning generic and sinonasal specific quality of life (EQ-5D-5L, SNOT-20-GV) showed an at least significant improvement for both dimensions (Table 2). In contrast to this there was no significant change over time for any of those dependent variables that were assessed by means of the WatchPAT200 (AHI, average snoring intensity, percent of sleeping time with a snoring intensity > 45 dB).

Additional analyses conducted separately for patients who at study enrollment did not show any problems regarding apneas or hypopneas during sleep (AHI < 5; *n* = 11) and those showing at least a mild sleep-related breathing disorder (AHI ≥ 5; *n* = 20) did not result in findings different from those reported for the total sample.

## Discussion

In this discussion, we will address several points, namely

- Defining the anatomy of the nasal valve.
- Interventions that address congestion at the nasal valve and their relative efficacy.
- Whether snoring be reduced by nasal valve surgery.
- Using objective versus subjective measures of effect for nasal congestion.

The inner nasal valve is bounded by the angle between the caudal border of the upper lateral cartilages (ULC) and dorsal septum. The external nasal valve extends from the nasal entrance to the INV and is bounded by the alar rim, the lower lateral cartilages (LLC), alar lobule and nasal sill inferiorly [18, 19].

Physiologically, the INV is the narrowest part of the nose and thus the location of the maximum air flow resistance. According to Hagen–Poiseuille’s law, the volume of airflow is directly proportional to the pressure difference and to the 4th power of the inner radius. This means that even the smallest increase in the area of the INV results in large reduction in the resistance of the nasal cavity and a noticeable improvement in the nasal airflow.

When air flows through a narrowed region it accelerates with a resulting reduction in pressure as described by the Bernoulli principle. In an unstable nasal valve, this has the effect of reducing or even closing the opening. Stabilizing this region can reduce the Bernoulli effect and improve nasal breathing.

Anomalies within the NVA are often responsible for nasal obstructions. The importance of the angle between the caudal boundary of the ULC and the nasal septum as a fundamental feature of the INV is immortalized in the literature and remains a key concept in the planning of surgical intervention. In fact, the NVA has a complex geometry and needs to be examined thoroughly and objectively before any INV surgery is proposed [20].

However, the fact that well-chosen surgical interventions such as septoplasty, turbinate reduction and spreader grafts, or combinations thereof, can improve nasal breathing is well established. Rhee et al. [5] in a literature review of 20 heterogeneous publications involving 643 patients undergoing various types of nasal surgery who were evaluated using NOSE score showed that average improvements of − 42 points were achieved post surgery.

The Vivaer system works via the principle of using targeted low-energy radiofrequency to heat and reshape the tissues of INV to create more space in the nose. Wong et al. were able to show in an experimental setting that it is possible to reshape cartilage with electrical energy and that this treated cartilage also retains its new shape with no significant weakening [21]. In a further study, they showed that it is possible to achieve the same effect using electromagnetic radiation without causing excessive heating of the tissues [22]. Keefe at al. used RF-generated heat to reform porcine nasal septal cartilage which retained its shape after 14 days [23]. In addition, this group showed that chondrocytes in the cartilage survive this form of RF reshaping. The prerequisites for efficacy at tissue shaping with the preservation of cellular and structural integrity are the optimization of electrode geometry, generator frequency, power and heating time.

The use of RF energy to stabilize the cartilage of the INV is not without precedent: Seren successfully used low-energy RF introduced to the ULC to stabilize the nasal valve (Celon-ProBreath; Celon AG Medical Instruments, Berlin, Germany) [24]. However, this procedure was more invasive as the access to the cartilage was created by an incision and the RF introduced via inserted probes with the incision closed with a suture after the intervention. The improvement in symptoms of nasal obstruction after treatment compared with before treatment was evaluated using the visual analog scale (VAS). Each of the 29 patients in this study showed a significant improvement in the severity of obstruction scores in the VAS 4 months after treatment. In another study, RF stabilization therapy (*n* = 7) was compared with the Bone-Anchored Suspension technique (BAST) (*n* = 6) for lateral nasal wall collapse [25]. Both groups benefited from the treatment and improved in NOSE score post-treatment compared pre-treatment with no significant difference between the two groups.

The results of the present study also showed a significant improvement in the NOSE score post-treatment vs. pre-treatment in 80% of patients. In slightly more than one-third of the cases, there was even an improvement of 2° or 3° of severity in the NOSE score. The improvement in the NOSE score by − 35 in median shown here is comparable to the results generally achieved by more invasive surgical interventions as shown by Rhee even given the exacting nature of the ITT analysis applied in this study [6].

A subanalysis of the improvement in the NOSE score indicates that in general the Vivaer procedure showed the greatest benefit to the more congested individuals. This is not unexpected given Hagen–Poiseuille’s law: those patients with the narrowest INV will receive a proportionally greater benefit with the addition of just a little extra space in the lumen.

The significant improvement in the snoring and sleep-related SOS questionnaire is also reflected elsewhere in the literature albeit that the evidence is less clear cut. Although Virkkula et al. showed no effect of nose surgery on snoring [26], other observational studies have shown that nasal congestion has a considerable impact on snoring and daytime sleepiness [27]. Similarly, Leitzen found no significant correlation between anatomical nasal obstruction and the severity of obstructive sleep apnoea in their study [28]. In contrast, a recently published meta-analysis on the efficacy of isolated nasal surgery in OSA patients showed a significant improvement in both the Epworth Sleepiness Scale and RDI [29]. In the present study, the SOS score shows a 90-day improvement over baseline of 27 points (from 66.6 to 41.6). This marked improvement of the subjective SOS scores in this study is in stark contrast to the lack of improvement in the objective WatchPAT200 data and this will be discussed shortly.

The significant improvement of patients’ Quality of Life as show for both the SNOT-20-GAV and EQ-5D questionnaires is not unexpected for an effective technique to improve nasal breathing. Patients with problems with nasal breathing have numerous other problems such as the need to blow the nose, sneezing, facial pain, loss of productivity, sleep disturbance, snoring, restlessness and other issues which lead to a decreased quality of life [3, 30]. Improvement in one or more of these areas can be expected to be reflected in a QoL questionnaire.

The procedure was shown to be well tolerated with all patients treated under only local anesthetic. No complications occurred with any patient and with no change in the external appearance of the nose. There was no incapacity to work caused by the treatment and all patients were able to return to their daily activities. Other observations from the study showed that the total procedure time was generally under 15 min.

From the literature, it is apparent that there is, as yet, no objective measuring instrument which provides reproducible results regarding the degree and nature of nasal obstruction or which closely correlated with both patient sensations of breathing and the results of macroscopic and endoscopic examinations [31]. In the literature, large differences are reported between objective and subjective measurements of nasal obstruction [32–34]. Even the application of reformatted computer tomography to assess the INV proves inferior to the combination of physical examination of the nose and patient symptoms [35].

This trend of objective instrumentation being unable to measure subjective improvement reported by the patient or their bedpartners is reflected in this study with the measurements recorded using the WatchPAT200 system. In this case, one speculates as to whether the variability of sleep quality that people experience on a night-to-night basis generates too much noise in the data for any underlying trend to be statistically isolated without a very large increase in the quantity of data collected. This remains a subject for further investigation.

Computational fluid dynamics on digital nasal models could possibly provide objective data in the future. Until then the physical examination with a Cottle maneuver, the use of a validated questionnaire such as the NOSE Score and, last but not least, the experience of the nasal surgeon remains the gold standard for the positioning of surgical intervention.

The intervention performed in this study targets the upper anatomical structures of the INV which leads to the conclusion that most widening occurs near the intersection of the ULC with the nasal septum. This approach appears to represent the greatest improvement in obstruction with minimal surgical effort [36].

### Strengths and limitations of the study

The inherent limitation of this study is certainly the absence of a placebo group (non-treatment group) and the short follow-up period (average of 3 months). A further improvement to the study would be the substitution of the Epworth Sleepiness Scale for the SOS questionnaire which suffers some difficulties in administration and interpretation.

A strength of the study lies in the prospective study design since many studies investigating the effectiveness in the improvement of nasal breathing are retrospective studies (16–20). A further strength of the study is the use of validated questionnaires such as NOSE and SNOT 20; other studies only have a VAS as the measuring instrument. Worth mentioning is the statistical evaluation of the measurement results with ITT analysis. It is certainly no disadvantage that only two surgeons performed all assessments.

## Conclusions

In summary, the results of this study collected with validated measuring instruments show that low energy radiofrequency intranasal remodeling treatment to enlarge collapsing or narrow nasal valve areas is significantly effective. The patients with the most severe complaints benefitted most from the treatment. The therapy is also safe and well tolerated and as an outpatient procedure offers a benefit to the healthcare system.

## References

[1] Bugten V, Nilsen AH, Thorstensen WM, Moxness MH, Amundsen MF, Nordgård S (2016). Quality of life and symptoms before and after nasal septoplasty compared with healthy individuals. BMC Ear Nose Throat Disord.

[2] Lugaresi E, Cirignotta F, Coccagna G, Piana C (1980). Some epidemiological data on snoring and cardiocirculatory disturbances. Sleep.

[3] Goldman ND, Alexander R, Sandoval LF, Feldman SR (2017). Nasal valve reconstruction using a titanium implant: an outcomes study. Craniomaxillofac Trauma Reconstr.

[4] Stewart MG, Smith TL, Weaver EM, Witsell DL, Yueh B, Hannley MT, Johnson JT (2004). Outcomes after nasal septoplasty: results from the Nasal Obstruction Septoplasty Effectiveness (NOSE) study. Otolaryngol Head Neck Surg.

[5] Lipan MJ, Most SP (2013). Development of a severity classification system for subjective nasal obstruction. JAMA Facial Plast Surg.

[6] Rhee JS, Sullivan CD, Frank DO, Kimbell JS, Garcia GJM (2014). A systematic review of patient reported nasal obstruction scores: defining normative and symptomatic ranges in surgical patients. JAMA Facial Plast Surg.

[7] Gliklich RE, Wang PC (2002). Validation of the snore outcomes survey for patients with sleep-disordered breathing. Arch Otolaryngol Head Neck Surg.

[8] Baumann I (2009). Validierte Lebensqualitätsmessinstrumente zur Anwendung bei. Patienten mit chronischer Rhinosinusitis: HNO.

[9] Baumann I, Blumenstock G, DeMaddalena H, Plinkert PK, Piccirillo JF (2007). Validierung des Sino-Nasal Outcome Test-20 German Adapted Version (SNOT-20 GAV) zur Messung der Lebensqualität bei Patienten mit chronischer Sinusitis. HNO.

[10] Baumann I, Plinkert PK, De Maddalena H (2008). Entwicklung einer Bewertungsskala für den Sino-Nasal Outcome Test 20 German Adapted Version (SNOT-20 GAV). HNO.

[11] Piccirillo JF, Merritt MG, Richards ML (2002). Psychometric and clinimetric validity of the 20-Item Sino-Nasal Outcome Test (SNOT-20). Otolaryngol Head Neck Surg.

[12] Brooks R (1996). EuroQoL: the current state of play. Health Policy.

[13] Hinz A, Kohlmann T, Stöbel-Richter Y, Zenger M, Brähler E (2014). The quality of life questionnaire EQ-5D-5L: psychometric properties and normative values for the general German population. Qual Life Res.

[14] Pang KP, Gourin CG, Terris DJ (2007). A comparison of polysomnography and the WatchPAT in the diagnosis of obstructive sleep apnea. Otolaryngol Head Neck Surg.

[15] Yuceege M, Firat F, Demir A, Ardic S (2013). Reliability of the Watch-PAT 200 in detecting sleep apnea in highway bus drivers. J Clin Sleep Med.

[16] Blair RC, Higgins JJ (1980). A comparison of the power of Wilcoxon’s rank-sum statistic to that of student’s t statistic under various nonnormal distributions. J Educ Stat.

[17] Blair RC, Higgins JJ (1980). Comparison of the power of the paired samples t test to that of Wilcoxon’s signed-ranks test under various population shapes. Psychol Bull.

[18] Cervelli V, Spallone D, Davide Bottini J, Silvi E, Ventile P, Curcio B, Pascali M (2009). Alar Batten Cartilage graft: treatment of internal and external nasal valve collapse. Aesth Plast Surg.

[19] Ballert JA, Park SS (2008). Functional considerations in revision rhinoplasty. Facial Plast Surg.

[20] Miman MC, Deliktaş H, Ozturan O, Toplu Y, AkarçayM (2006). Internal nasal valve: revisited with objective facts. Otolaryngol Head Neck Surg.

[21] Manuel CT, Foulad A, Protsenko DE, Sepehr A, Wong BJ (2010). Needle electrode-based electromechanical reshaping of cartilage. Ann Biomed Eng.

[22] Manuel CT, Foulad A, Protsenko DE, Hamamoto A, Wong BJ (2011). Electromechanical reshaping of costal cartilage grafts: a new surgical treatment modality. Laryngoscope.

[23] Keefe MW, Rasouli A, Telenkov SA, Karamzadeh AM, Milner TE, Crumley RL, Wong BJ (2003). Radiofrequency cartilage reshaping. Efficacy, biophysical measurements, and tissue viability. Arch Facial Plast Surg.

[24] Seren E (2009). A new surgical method of dynamic nasal valve collapse. Arch Otolaryngol Head Neck Surg.

[25] Weissman JD, Most SP (2015). Radiofrequency thermotherapy vs bone-anchored suspension for treatment of lateral nasal wall insufficiency: a randomized clinical trial. JAMA Facial Plast Surg.

[26] Virkkula P, Bachour A, Hytönen M, Salmi T, Malmberg H, Hurmerinta K, Maasilta P (2006). Snoring is not relieved by nasal surgery despite improvement in nasal resistance. Chest.

[27] Georgalas C (2011). The role of the nose in snoring and obstructive sleep apnoea: an update. Eur Arch Otorhinolaryngol.

[28] Leitzen KP, Brietzke SE, Lindsay RW (2014). Correlation between nasal anatomy and objective obstructive sleep apnea severity. Otolaryngol Head Neck Surg.

[29] Ishii L, Roxbury C, Godoy A, Ishman S, Ishii M (2015). Does nasal surgery improve OSA in patients with nasal obstruction and OSA? A meta-analysis. Otolaryngol Head Neck Surg.

[30] Chambers KJ, Horstkotte KA, Shanley K, Lindsay RW (2015). Evaluation of improvement in nasal obstruction following nasal valve correction in patients with a history of failed septoplasty. JAMA Facial Plast Surg.

[31] Valsamidis K, Titelis K, Rachovitsas D, Konstantinidis I, Markou K, Triaridis S (2018). Long-term evaluation of nasal septoplasty followed by inferior turbinate cauterization for the treatment of nasal obstruction using objective and subjective methods. Int Arch Otorhinolaryngol.

[32] Dinis PB, Haider H (2002). Septoplasty: long-term evaluation of results. Am J Otolaryngol.

[33] Toyserkani NM, Frisch T, Von Buchwald C (2013). Postoperative improvement in acoustic rhinometry measurements after septoplasty correlates with longterm satisfaction. Rhinology.

[34] André RF, Vuyk HD, Ahmed A, Graamans K, Nolst Trenité GJ (2009). Correlation between subjective and objective evaluation of the nasal airway. A systematic review of the highest level of evidence. Clin Otolaryngol.

[35] Bloom JD, Sridharan S, Hagiwara M, Babb JS, White WM, Constantinides M (2012). Reformatted computed tomography to assess the internal nasal valve and association with physical examination. Arch Facial Plast Surg.

[36] Dolan RW (2010). Minimally invasive nasal valve repair: an evaluation using the NOSE scale. Arch Otolaryngol Head Neck Surg.

